# Anti-Scar Effects of Micropatterned Hydrogel after Glaucoma Drainage Device Implantation

**DOI:** 10.34133/research.0561

**Published:** 2025-01-22

**Authors:** Yiling Han, Qiangwang Geng, Aimeng Dong, Menglu Jiang, Jingyi Ma, Wulian Song, Pan Fan, Yuanyuan Li, Jiawen Gao, Fenghua Zhang, Jinsong Leng, Huiping Yuan

**Affiliations:** ^1^ Department of Ophthalmology, The Future Medicine Laboratory, The Second Affiliated Hospital of Harbin Medical University, Harbin 150086, People’s Republic of China.; ^2^ Centre for Composite Materials and Structures, Harbin Institute of Technology (HIT), Harbin 150080, People’s Republic of China.

## Abstract

Excessive fibrosis is the primary factor for the failure of glaucoma drainage device (GDD) implantation. Thus, strategies to suppress scar formation in GDD implantation are crucial. Although it is known that in implanted medical devices, microscale modification of the implant surface can modulate cell behavior and reduce the incidence of fibrosis, in the field of ophthalmic implants, especially the modification and effects of hydrogel micropatterns have rarely been reported. Here, we designed the patterned gelatin/acrylamide double network hydrogel and developed an innovative GDD with micropattern to suppress inflammatory and fibroblast activation after GDD implantation. Pattern topography suppressed F-actin expression and mitigated actin-dependent nuclear migration of myocardin-related transcription factor A (MRTF-A) during the proliferative phase after GDD implantation. Ultimately, the expression of α-smooth muscle actin (α-SMA), a key fibrosis-related gene product, was suppressed. Moreover, the modified GDD effectively controlled intraocular pressure (IOP), mitigated fibrous formation, and remodeled extracellular matrix (ECM) collagen distribution in vivo. Therefore, the novel GDD with surface patterning interventions provides a promising strategy to inhibit scar formation after GDD implantation and raise the efficacy of GDD implantation.

## Introduction

Glaucoma, the second most common cause of permanent blindness globally, comprises progressive retinal ganglion cell degeneration and subsequent optic nerve loss [1]. Filtration surgery, such as glaucoma drainage device (GDD) implantation, is commonly employed as a main method of surgical intervention for advanced glaucoma and refractory glaucoma [2]. However, excessive fibrosis surrounding GDD impedes aqueous humor filtration, precipitating surgical failure [3]. In the past years, numerous methodologies have been investigated to suppress scar formation such as the application of antimetabolites during surgery [4], drug delivery systems capable of sustained mitomycin C (MMC) release [5–8], and exploration of alternative inert materials to supplant silicon plates [9–11]. However, these strategies have limited effects on blocking or delaying the development of scar formation, and the issue of postoperative scarring in GDD implantation remains a marked challenge.

The proliferative phase of scar formation, characterized by fibroblast proliferation, collagen production, and extracellular matrix (ECM) generation, is critical in wound healing [12]. Thus, exploring innovative approaches for fibroblast activation and suppressing inflammation after GDD implantation are promising to relieve postoperative scarring. Research has confirmed that promoting the M2 macrophage phenotype at the material interface importantly reduces fibrous capsule formation around the implant [13,14].

Surface topographies, designed with regular geometries, have been demonstrated to influence cell behaviors, including cell adhesion, proliferation, and differentiation [15–19]. Regulating surface characteristics is a general tactic to improve their bio-integration potential and reduce fibrosis incidence in implanted medical devices such as those used in skin wound healing and breast augmentation [16,20–22]. Implants with roughness of 2 to 5 μm can validly inhibit foreign body reaction (FBR) and fibrosis [16,23]. Notably, microscale pits, hexagonal patterns, and squared topographies have shown promise in mitigating inflammatory FBRs, controlling fibroblast adhesion, and decreasing the activation of α-smooth muscle actin (α-SMA)-positive myofibroblasts [16,19,20,24]. However, this innovation strategy has yet to be expanded into ophthalmic clinical practice.

Water-based hydrogels, due to their exceptional biocompatibility, flexibility, and low cytotoxicity, have extensive applications in biomedicine, including drug delivery, preventing postoperative adhesion and so on [25,26]. In this study, we developed a gelatin/acrylamide double network (DN) hydrogel with tunable surface microstructures, enabling precise regulation of cellular structures. We investigate the effects of a regular grooved micropattern on cell morphology, polarization, cytokine production, and modulation of the actin–myocardin-related transcription factor (MRTF) signaling axis. Further, we implanted the modified GDD made of hydrogel with optimal groove width into rabbit eyes and investigated the inhibitory effects of micropattern on bleb scarring in vivo.

## Results

### Characterization of hydrogel

#### Preparation of patterned PG hydrogel

**Fig. 1.**
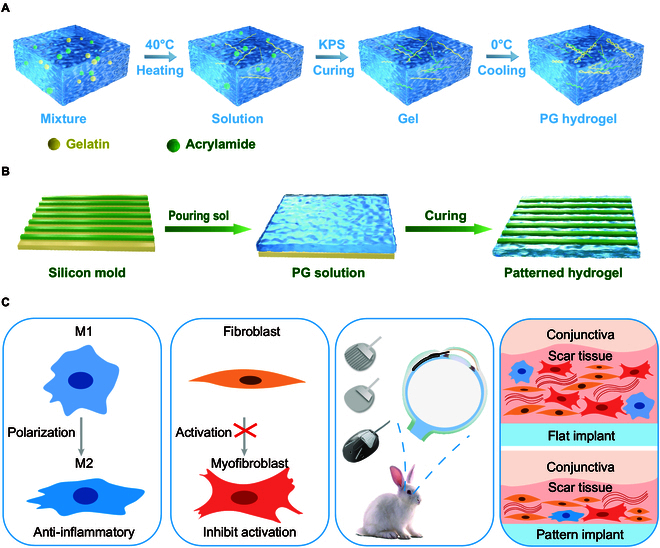
The synthesis process of patterned PG hydrogel. (A) Synthesis mechanism of PG hydrogel. (B) Schematic diagram of patterned hydrogel synthesis. (C) Multifunction of patterned hydrogel including anti-inflammatory, promoting proliferation, and preventing scarring.

The synthesis mechanism of polyacrylamide/gelatin (PG) hydrogels is illustrated in Fig. 1A. DN hydrogels, two intertwined and mutually cross-linked polymer networks, typically consist of a highly cross-linked rigid network in combination with a loosely cross-linked and flexible soft network. The first network provides mechanical strength, while the second network enables energy dissipation during large deformations, thereby ensuring high strength and excellent stretchability of the hydrogel. Gelatin, PG, and *N*,*N*′-methylenebisacrylamide (MBA) are dissolved in deionized water, and the mixture is heated to 40 °C, allowing the gelatin to dissolve and form the first network completely. Subsequently, potassium persulfate (KPS) is added to the solution. The temperature is then raised to 60 °C, leading to the formation of the second network through the polymerization of acrylamide. Finally, the temperature is lowered to 0 °C, causing the gelatin to adopt a helical structure. The process for preparing patterned PG hydrogels is depicted in Fig. 1B. The prepared PG solution is poured onto silicon wafers with prefabricated surface microstructures. The system is then cured at 60 °C, followed by a 30-min incubation at 0 °C. Finally, the patterned PG hydrogel is peeled off from the silicon wafer, completing the fabrication process.

#### Tensile properties

**Fig. 2.**
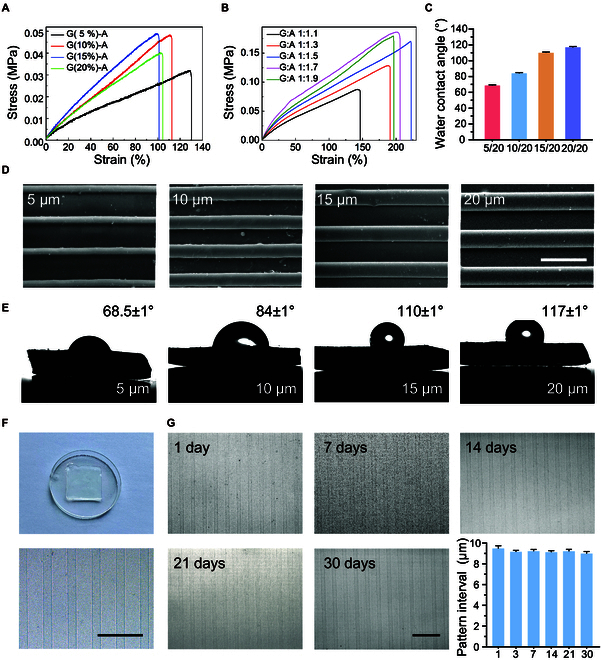
(A) Stress–strain curves of PG hydrogels containing different amounts of gelatin. (B) Stress–strain curves of PG hydrogels with varying levels of acrylamide. (C) Water contact angle data of PG hydrogel. (D) SEM of the patterned PG hydrogels with different stripe-to-spacing ratios (5/20, 10/20, 15/20, and 20/20). (E) Water contact angle diagram of PG hydrogels. (F) Images of produced patterned hydrogel and its magnified surface (10 μm). (G) Optical images and variation of pattern interval of hydrogels after immersion in PBS for 30 d. Scale bars, 50 μm.

It was essential to assess the mechanical properties of PG hydrogels prepared with different ratios to satisfy the specific requirements of ophthalmic applications. Figure 2A presents a stress–strain curve for the PG hydrogel, where the acrylamide content is kept constant while varying the gelatin content. The PG hydrogel demonstrated its highest mechanical strength (0.049 MPa) at a gelatin concentration of 15%. Maintaining the gelatin content at 15%, we introduced different acrylamide proportions and measured the stress–strain curve of the PG hydrogel, as illustrated in Fig. 2B. The mechanical strength reached its peak, at 0.186 MPa, when the gelatin-to-acrylamide ratio was 1:1.7. Therefore, we prepared the patterned PG hydrogel when the gelatin content was 15% and the ratio of gelatin to acrylamide was 1:1.7.

#### Performance test of patterned hydrogel

We initially tested surface morphology on the prepared patterned PG hydrogels, fabricated with stripe-to-spacing ratios of 5/20, 10/20, 15/20, and 20/20, respectively. The patterned hydrogel was taken after the hydrogel had swollen, so the image was the clearer picture that could be obtained. The scanning electron microscopy (SEM) observation in Fig. 2D shows that the patterned PG hydrogel demonstrated a regular striped structure with clear edges. The water contact angle increases with increased stripe width (Fig. 2C and E), indicating a decrease in hydrophilicity. Surfaces with a hydrophobic nature (contact angle > 90°) could trigger biomolecule adhesion and proliferation [27]. Contact angles varying from 70° to 80° minimize protein adsorption, and the intensity of FBR has been identified as optimal for in vitro fibroblast culture [28–30]. Given these superiorities, the 10/20 pattern PG hydrogel was considered an attractive material for manufacturing GDDs. To evaluate the stability of patterned PG hydrogels, we immersed them in phosphate-buffered saline (PBS) for 30 d. Figure 2G shows that the strip structure remained clear and intact, demonstrating the good stability of patterned hydrogel and its suitability for extended implantation.

### Biocompatibility in vitro

**Fig. 3.**
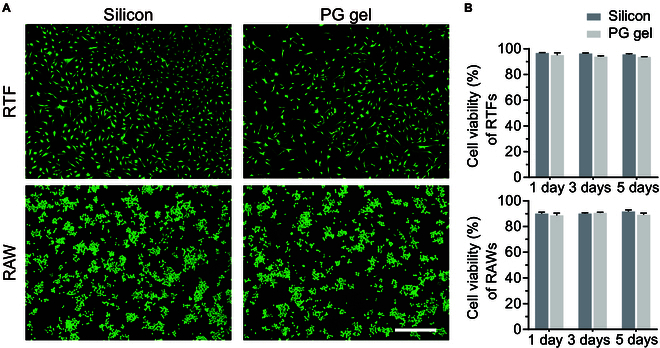
The biocompatibility and cytotoxicity in vitro. (A) Live/dead staining of RTFs and RAWs co-incubated with AGV silicon and PG hydrogels. Scale bar, 250 μm. (B) CCK-8 assay detected cell viability at 1, 3, and 5 d. Scale bar, 250 μm.

The biocompatibility of PG hydrogels was assessed by live/dead staining and compared Ahmed glaucoma valve (AGV; a type of GDD commonly used in clinical practice) silicon. As illustrated in Fig. 3A, after 5 d of co-incubation, RAW264.7 mouse monocyte macrophage disease cells (RAWs) and rabbit tenon fibroblasts (RTFs) exhibited intense green and minimal red fluorescence, indicating the excellent cytocompatibility of the PG hydrogel (Fig. 3B). Meanwhile, the Cell Counting Kit-8 (CCK-8) assay showed that both control and PG hydrogel exhibited high cell viability. These results indicated that the PG hydrogel had good biocompatibility and low cytotoxicity could provide an appropriate growth environment for relevant cells.

### Morphology and phenotype transition of RAWs induced by micropattern

**Fig. 4.**
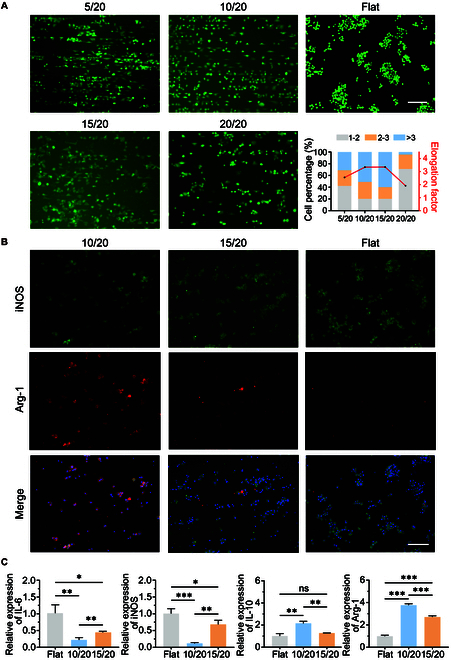
Pattern topography regulates macrophage morphology and phenotype in vitro. (A) Images and elongation factor of RAWs on PG hydrogels with different micropatterns. (B) Fluorescence images of RAWs on 10/20, 15/20, and flat gel immunostained for iNOS (M1 macrophage marker, green), Arg-1 (M2 macrophage marker, red), and nuclei [4′,6-diamidino-2-phenylindole (DAPI), blue]. Scale bars, 100 μm. (C) Expression of IL-6, iNOS, IL-10, and Arg-1 in RAWs cultured on hydrogel. Data are shown as fold change over glyceraldehyde-3-phosphate dehydrogenase (GAPDH) (2^−ΔΔCt^). Scale bars, 100 μm.

A direct contact assay was conducted between cells and patterned hydrogels to assess the anti-inflammatory potential of topographical patterns. As depicted in Fig. 4A, cells on flat surfaces typically assumed a roughly circular shape, whereas those on patterned hydrogels were compelled to elongate. The elongation factor is the ratio of the longest to the shortest axis of the cell nucleus, reaching approximately 3.5 in the 10/20 and 15/20 pattern groups, in contrast to a ratio of about 1 for those on flat surfaces. These observations suggest that micropatterned surfaces promote cell adhesion and induce macrophage elongation.

Previous studies showed that macrophage elongation induced by micropatterns enhances the anti-inflammatory phenotypic marker expression and is relevant to groove size [18,31–33]. To determine the most effective paralleled pattern topologies on macrophage polarization state, macrophages were seeded on flat, 10/20, and 15/20 PG hydrogels, respectively. The 10/20 and 15/20 groups demonstrated anti-inflammatory polarization compared to the control group (Fig. 4B), implying that macrophages undergo spontaneous differentiation on patterned hydrogels.

Reverse transcription polymerase chain reaction (RT-PCR) quantitative analysis in Fig. 4C corroborated that cells cultured on 10/20 hydrogels displayed a significantly lower expression in genes associated with pro-inflammatory molecules [interleukin-6 (IL-6) and inducible nitric oxide synthase (iNOS)] and an increased expression in anti-inflammatory molecules (IL-10 and Arg-1). This suggested that 10/20 hydrogels may induce macrophage polarization toward an M2 phenotype to mitigate inflammation. To sum up, micropattern modulated macrophage elongation and polarization.

### Morphological and functional transition of RTFs induced by micropattern

**Fig. 5.**
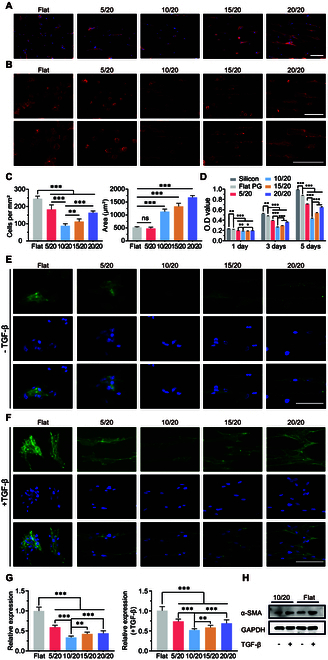
Pattern topography regulates fibroblast morphology and activation in vitro. (A) Images of RTFs on PG hydrogels after culturing for 72 h. (B) Immunofluorescence staining of F-actin. The illustration below shows the magnified cell morphology. (C) Statistical analysis of cell density and cell spreading area. (D) Optical density (O.D) values of RTFs on silicon, flat, 5-μm, 10-μm, 15-μm, and 20-μm pattern gels after 1, 3, and 5 d of culture. (E) Images of RTFs adhering to the pattern topography after culturing for 72 h with and (F) without profibrotic TGF-β. RTFs were stained for α-SMA (green) and nuclei (blue). (G) Statistical results of α-SMA relative expression. (H) Western blotting analysis of α-SMA expression of RTFs on flat hydrogel and 10/20 hydrogel before and after TGF-β stimulation. Scale bars, 100 μm.

Cell morphology on patterned surfaces was evaluated in Fig. 5A. Cells exhibited a rounded morphology on flat surfaces. Notably, cells maintained a round shape on 5/20 hydrogel, whereas an increase in pattern width resulted in a transition to a spindle shape. Cells on flat surfaces appeared randomly oriented, spreading out in all directions, while those on patterned surfaces adhered along the grooves, exhibiting obvious alignment growth. Pattern topographies curtailed RTF adhesion and cell morphology dictated by the pattern width. Meanwhile, RTFs showed a larger spreading area and a decreased cell density when cultured on patterned hydrogel (Fig. 5C). Furthermore, RTFs on the 10/20 hydrogel exhibited minimal cell adhesion and proliferation at all time points (Fig. 5D), indicating its anti-proliferative function. Typical fluorescence images of filamentous actin (F-actin), a critical component of the cytoskeleton, are shown in Fig. 5B. A stronger actin stress fiber was observed on cells cultured on flat hydrogels, while RTFs on 10/20 hydrogels existed with minimal F-actin expression.

Myofibroblast is a smooth muscle-like contractile cell derived from fibroblast and is considered crucial in initiating the implant-induced fibrotic response. Its activation induces dense scar formation [34]. To evaluate the impact of micropattern on myofibroblast activation, α-SMA expression was quantified. Less α-SMA expression levels existed in RTFs on pattern hydrogels compared to the flat group (Fig. 5E). Further quantitative measurement demonstrated that α-SMA expression in RTFs on 10/20 surfaces was minimal, suggesting its potential to mitigate fibroblast activation. Implants are exposed to an inflammatory environment with high circulating transforming growth factor-β (TGF-β) levels after in vivo implantation [35]. We treated RTFs with TGF-β to mimic a profibrotic environment in vitro (Fig. 5F). TGF-β mildly elevated α-SMA expression of cells on 10/20 micropattern (Fig. 5G). Western blot analysis corroborated that 10/20 micropattern could reduce α-SMA expression no matter whether TGF-β was stimulating (Fig. 5H and Fig. S1). Overall, our results demonstrated that the 10/20 pattern could inhibit fibroblast activation even in a profibrotic environment.

**Fig. 6.**
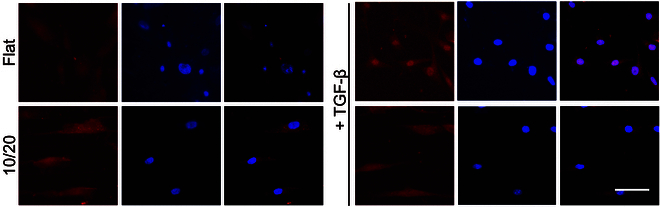
RTFs were stained for MRTF-A (red) and nuclei (blue) to observe the regulation of MRTF-A localization by patterned surfaces, both with and without TGF-β stimulation. Scale bar, 50 μm.

To investigate the mechanisms by which patterned hydrogel inhibits fibroblast activation, we examined MRTF-A expression in both normal and pro-fibrotic environments and explored the relationship between pattern topography and the activation of the actin–MRTF signaling pathway. Nuclear accumulation of MRTF-A was unobserved in neither flat nor patterned groups. Subsequently, when RTFs were treated with the pro-fibrotic factor TGF-β, nuclear translocation of MRTF-A remained suppressed on the 10/20 patterned surface; in contrast, the nucleus expression of the positive performance of MRTF-A was detected on the flat group (Fig. 6).

### In vivo measurements

**Fig. 7.**
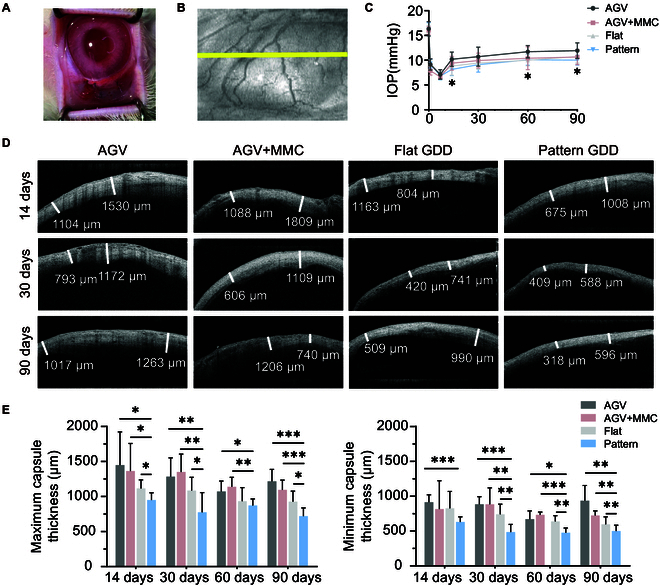
In vivo pattern PG implantation. (A) Image of the process of GDD implantation. (B) AS-OCT scan position (yellow line). (C) IOP measurements in the days 0, 1, 7, 14, 30, 60, and 90. (D) AS-OCT images of the filter bleb. (E) Maximum and minimum capsule thickness of filter bleb.

Given the remarkable in vitro results, the 10/20 pattern hydrogel was chosen to fabricate into GDDs. To determine its anti-fibrotic effect in vivo, we implanted AGV, flat GDD, and 10/20 pattern GDD to subconjunctival space (Fig. 7A). Photographs of the fabricated GDDs are shown in Fig. S2. Clinically, a stable filtration pathway is generally established within 3 months after surgery [5]. We monitored filter bleb by intraocular pressure (IOP) measurements and Anterior segment optical coherence tomography (AS-OCT). There were no instances of infection or other complications such as endophthalmitis, tube block, implant exposure, or filtering bleb leakage during the postoperative period. Conjunctival edema was observed in all groups postoperatively and gradually subsided within 2 weeks.

IOP rapidly decreased postoperatively and gradually climbed up. Repeated-measures analysis revealed that patterned GDD maintained a lower IOP level and exhibited the longest duration of sustained low IOP (Fig. 7C), indicating the establishment of a functional filter bleb. A durable, sufficiently permeable bleb is crucial for safe and successful IOP reduction in glaucoma surgery. Further evaluation of filtering bleb would help to assess postoperative scar formation. We used AS-OCT to observe bleb morphology. The yellow line showed AS-OCT scanning position (Fig. 7B). The transverse image of a filtering bleb was composed of a highly reflective bubble wall and hypo-reflective internal liquid-filled cavity. The bleb wall displayed a relatively regular surface (Fig. 7D). To eliminate the influence of minor differences in implant morphology on the thickness of the filtering bleb walls, we took AS-OCT images of the filter bleb on the day after operation (0 d). There was no statistically significant difference among the AGV, flat GDDs, and pattern GDDs groups on the day after operation, but the maximum bleb wall thickness in the AGV + MMC group was significantly thicker compared to other groups (Fig. S2). This phenomenon is due to the irritative effect of intraoperative MMC use on the local conjunctiva, causing localized conjunctival edema. The long-term AS-OCT quantitative measurement displayed that the filter bleb was significantly thinner in the pattern group in all periods (Fig. 7E) and showed a positive correlation with IOP.

### Histological examination

To evaluate the modality of pattern topography to the inflammatory response, we examined inflammatory cell aggregation around the implant 4 d after implantation. As shown in Fig. S3, diffuse inflammatory cell infiltration was observed around the AGV and the AGV + MMC group through H&E staining at 4 d after operation, whereas almost no inflammatory cell was detected around the pattern group. Moreover, further staining of inflammation-related markers demonstrated a significant expression of the pro-inflammatory cytokine CD86 in the tissue surrounding the implants of the AGV group. These finding suggest the in vivo anti-inflammatory function of the patterned implants.

**Fig. 8.**
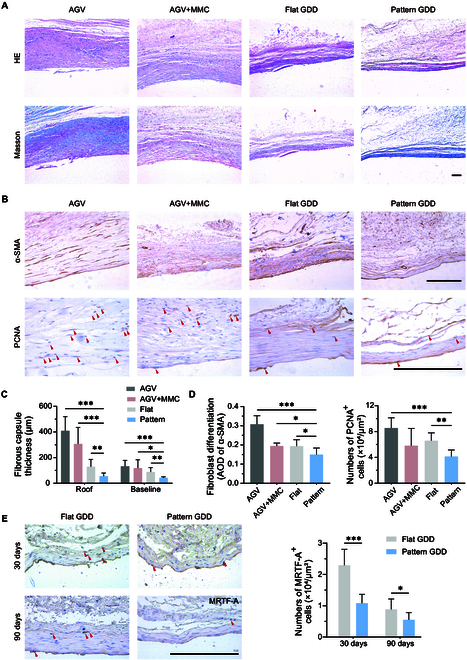
Histological and IHC graphics of the filter bleb. (A) Representative images stained with hematoxylin and eosin (H&E) and Masson staining. (B) Typical IHC images stained with α-SMA and PCNA. (C) Fibrous capsule thickness surrounding implants on their roof and baseline side. (D) Quantitative analysis of α-SMA and PCNA relative expression. (E) Typical IHC images stained with MRTF-A and analyzed MRTF-A nuclear positive expression at 30 and 90 d after operation. Scale bars, 100 μm.

Histological observations were performed at 3 months after operation. Pathological observation showed that collagenous fibrous tissue that accumulated around AGV was substantially denser and thicker than that in flat and pattern groups (Fig. 8A). The fibrous capsule thickness quantitative measurement showed a significant difference (*P* < 0.05) between AGV and pattern groups (Fig. 8C). Masson staining showed that collagen fiber deposition was disturbed. Collagen density was significantly reduced in the pattern group. The lower and looser fibrous tissue around pattern GDDs revealed a better aqueous humor drainage result.

Immunohistochemical (IHC) staining in the AGV group showed a notable α-SMA immune expression in the scar tissue surrounding AGV but a diminished α-SMA-positive signal in the pattern group (Fig. 8B), indicating that the pattern GDD can effectively inhibit fibroblast activation in vivo. Subsequent staining of the myofibroblast-rich layer allowed for the analysis of MRTF-A nuclear expression. The pattern group showed significantly lower nuclear MRTF-A levels compared to the flat group at 30 and 90 d after surgery (Fig. 8E). The level of cellular proliferation marker proliferating cell nuclear antigen (PCNA) expression was lower in the pattern group compared to other groups (Fig. 8D). These experimental results suggest that the patterned GDD potentially inhibits scar formation by curtailing the proliferation and activation of fibroblasts in vivo.

**Fig. 9.**
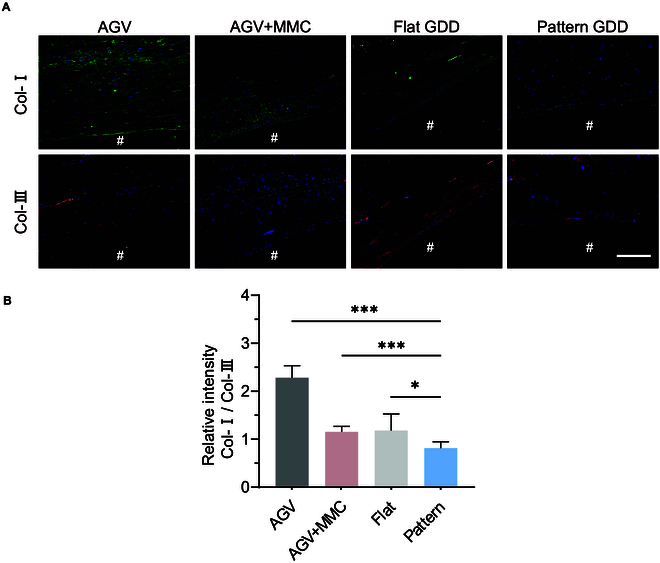
The distributions of different collagen types. (A) Typical immunofluorescence images stained with Col-I (green) and Col-III (red). # represents the location of the implant. (B) Relative intensity of Col-I/Col-III. Scale bar, 150 μm.

Immunofluorescent images were taken to evaluate the elements of the bleb capsule, stained for collagen I (Col-I) and collagen III (Col-III) within the fibrotic tissue (Fig. 9A). The collagenous fibrous tissue of AGV was composed of thicker and longer Col-I fibers, which were organized in a parallel arrangement. In contrast, the collagen network in the pattern group exhibited more Col-III fibers, which were shorter, thinner, and delicately arranged in reticular. The quantitative analysis revealed a significantly lower Col-I/Col-III ratio in pattern groups (Fig. 9B). This ratio verges on uninjured normal tissues, which corroborates the efficacy of patterned topography in limiting fibroblast activation and enhancing implant integration.

## Discussion

Failed IOP control at the mid and late stages after GDD implantation remains the main cause of blindness in glaucoma patients. To increase the surgery success rate of GDD implantation, our prior research coated AGV with opal shale microparticles (OS MPs) loading MMC, achieving sustained drug release over 18 d [6]. Additionally, we employed the electrostatic spinning technique to fabricate shape memory polylactic acid/polydioxanone (PLA/PPDO) fibers with the function of controlled drug release capabilities and then wrapped them around GDD plate body [7]. Two studies effectively controlled the further increase in IOP caused by scar formation around GDD to some extent but also highlighted the limitation of the transient effects of these drug-loading approaches. Our previous studies found that modulating GDD surface properties influences fibrosis formation. However, research on anti-scarring by modifying the physical characteristics of biomaterials comprising GDDs remains unexplored. Hence, we designed a regular grooved micropattern hydrogel to diminish fibrosis and enhance surgical success rates continuously.

DN hydrogels, comprising two mutually cross-linked polymer networks, typically consist of a highly cross-linked rigid network that imparts mechanical strength, combined with a loosely cross-linked and flexible soft network facilitating energy dissipation during deformations. This unique combination ensures exceptional tensile strength and remarkable stretchability of the hydrogel. The PG hydrogel was chosen as an ideal biomaterial for its excellent biocompatibility and mechanical properties. Gelatin, possessing lower antigenicity, is extensively utilized in biomedical applications. We developed a gelatin/acrylamide DN hydrogel with tunable surface microstructures, enabling precise regulation of cellular structures. In our study, the tensile strength of the PG hydrogel reached 186 kPa, meeting the demand of processes during surgical operation. We employed silicon wafers with surface microstructures to fabricate patterned PG hydrogels. The fabrication resulted in well-replicated and highly durable micropatterns, which retained their topographies in PBS for at least 30 d, indicating their suitability for extended implantation. Patterns added to the PG gels confer a hydrophilic surface, influencing the adhesion of proteins, platelets, and cells [28]. Contact angle varying from 70° to 80° has been identified as optimal for in vitro fibroblast culture [28–30]. The contact angle of the 10/20 pattern surface was 84 ± 1°, which was the closest to the optimal range for in vitro fibroblast culture among the groups in our study. Given these superiorities, the 10/20 pattern PG hydrogel was considered an attractive material for manufacturing GDDs.

Our current study suggests that simultaneously altering the macrophage anti-inflammatory phenotype expression and suppressing myofibroblast activation may be an appropriate approach to inhibit fibrosis formation comprehensively. Successful implant integration relies on a delicate equilibrium between predominant macrophage phenotypes. Promoting an early shift to M2 macrophage phenotype at the material interface may reduce fibrous capsule formation around implanted biomaterials [14,36]. Regulating macrophage phenotype and behavior may be an effective means to achieving this goal. Macrophages cultured on pattern gels were induced to elongate and enhance the anti-inflammatory marker expression. A obvious difference in macrophage accumulation around the implant interface between the pattern and AGV group was observed at 3 d after operation, confirming the anti-inflammatory efficacy of the micropattern.

The proliferative phase, characterized by fibroblast proliferation, collagen synthesis, and ECM generation, is another critical stage in wound healing [12]. The proliferation and differentiation of Tenon’s fibroblasts are the main cause of scarring after glaucoma surgery. Pattern topography inhibits RTF adhesion and proliferation and yields favorable modulation of the fibrosis inhibition through suppression of myofibroblast activation. Compared to flat surfaces, micropattern affected filopodia formation, reducing F-actin formation. This phenomenon can be interpreted as pattern topography alleviating the mechanical stress, leading to less desmosome and actin filament formation [37,38]. Actin cytoskeletal dynamics directly regulate MRTF-A expression. When cells are mechanically stimulated, integrin-mediated mechano-signaling facilitates the polymerization of G-actin into F-actin, which in turn releases MRTF-A from G-actin, allowing its translocation into the nucleus. Together with serum response factor (SRF), MRTF-A drives the transcription of profibrotic genes like connective tissue growth factor (CCN2) and ACTA2 (α-SMA) [39]. Nuclear recruitment of the mechanosensitive transcriptional coactivator MRTF-A and myofibroblast activation was inhibited on the patterned surface, regardless of the profibrotic environment, suggesting that topographical cues could override the chemical surface effects. We supposed that the patterned topography suppressed F-actin expression, thereby inhibiting the actin-dependent nuclear recruitment of MRTF-A and subsequently myofibroblast activation.

Histological examination indicated that the fibrosis thickness of pattern GDDs was significantly thinner than that in other groups. This outcome was comparable to formerly published studies on drug-loaded [6–8] or structurally optimized GDDs [10]. Compared with the use of MMC during the operation, the patterned GDDs have a better anti-scar effect. Additionally, we observed that several inflammatory cells accumulated around the flat implants, but this phenomenon was less pronounced in the patterned groups, probably because the pro-inflammatory macrophages that infiltrated around the implant were inhibited. The accumulation of contractile myofibroblasts ultimately increases tension, leading to a positive feedback loop and dense scar formation. A diminished α-SMA-positive signal and PCNA-positive cells were observed in the pattern group, indicating its efficiency in inhibiting myofibroblast activation and cellular proliferation in vivo. An increased Col-I/Col-III ratio in the AGV group results in wider and stiffer fibers. A stiffer ECM applies greater force to cells via focal adhesions, inducing higher cytoskeletal tension [40]. This mechanical signaling regulates the activation of fibroblasts to myofibroblasts. The Col-I/Col-III ratio of uninjured normal tissues such as skin, cardiac, and vascular tissues is approximately 2:1, whereas this ratio increases to 5:1 in scar tissues [41]. The Col-I/Col-III ratio in the pattern group corresponds to uninjured tissue verifying the efficacy of patterned topography in limiting fibroblast activation and enhancing implant integration.

**Fig. 10.**
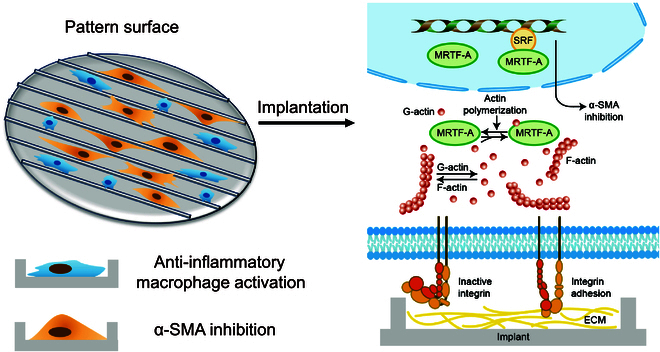
Proposed mechanism of PG hydrogel modified with surface patterning suppressed capsular fibrosis after GDD implantation.

Significant reductions in inflammatory cell accumulation and fibrosis formation seen around the pattern GDDs demonstrate improved functional outcomes. Pattern topography drives a suppression of fibroblast proliferation and differentiation that would be expected to have sustained effects for at least 90 d. Consequently, our results present a promising approach to leverage the immune and proliferative responses of the implant to support and enhance scarless tissue repair and maintain IOP and functional filter bleb. The possible antifibrotic mechanisms were as follows. First, pattern topography induces macrophage polarization toward an M2 phenotype to mitigate inflammation. Second, pattern topography provides topographic signals that modify fibroblast deposition and orientation and decrease mechanical stress on cells, suppressing F-actin expression, thereby inhibiting the actin-dependent nuclear translocation of MRTF-A, leading to reduced myofibroblast activation. Third, the lower Col-I/Col-III ratio revealed a soft and flexible filter bleb and lower cytoskeletal tension provided a wound-healing environment without excessive mechanical tension and fibrosis (Fig. 10). Our study here provides a promising strategy to inhibit scar formation after GDD implantation and raise the efficacy of GDD implantation.

## Materials and Methods

### Experimental design

We designed the patterned gelatin/acrylamide DN hydrogel and detected its microstructures, mechanical properties, hydrophilia, long-term stability, and biocompatibility. Then, we observed macrophage polarization, fibroblast adhesion, proliferation, and activation and explored mechanisms of suppressed myofibroblast activation relevant to micropattern. Finally, the novel GDD with micropattern was implanted into the rabbit’s eyes. Subsequently, the intraocular pressure and the thickness of the filter bleb wall were monitored at regular intervals postoperatively, and a histological examination was carried out 3 months following the surgical procedure.

### Materials

Acrylamide (99%), gelatin, and MBA were procured from Aladdin (Shanghai) Inc., and all reagents were used without the need for additional purification.

### Fabrication of PG hydrogel

The PG hydrogels were synthesized using a one-pot thermal curing method. Initially, the hydrogels were prepared with different gelatin concentrations, keeping the PG mass fraction constant. Gelatin was incorporated into the system at mass fractions of 5%, 10%, 15%, and 20%. The mixture was subsequently supplemented with 0.23 wt % KPS, 0.05 wt % MBA, and deionized water, and subjected to thermal curing at 60 °C for 1 h, followed by cooling at 0 °C for 30 min. After evaluating the mechanical properties, gelatin at a 15% mass fraction was chosen for further synthesis. With the gelatin mass fraction held constant, the gelatin-to-PG ratio was adjusted from 1:1.1 to 1:1.9. This procedure was replicated, introducing the required quantities of MBA, deionized water, and KPS, curing at 60 °C for 1 h, and then cooling at 0 °C for 30 min.

### Fabrication of patterned PG hydrogel

The preparation process of patterned PG hydrogels is similar to that of PG hydrogels. After selecting the appropriate ratio of gelatin to PG, gelatin, PG, and MBA are dissolved in a suitable amount of deionized water, and KPS is added (0.23 wt %). Then, the aforementioned solution is poured onto silicon wafers with different surface microstructures, where the ratio of stripes to spacing is set as 5/20, 10/20, 15/20, and 20/20, respectively. The gel solution on the silicon wafers is placed at 60 °C for 1 h and then refrigerated for half an hour. Subsequently, the patterned PG hydrogel is peeled off from the silicon wafer, resulting in the formation of patterned PG hydrogels.

### Characterization

#### SEM test

The surface microstructures were characterized using the Hitachi’s SU5000 SEM to examine the morphology of the patterned PG hydrogels, with an acceleration voltage of 20 kV, a working distance of 40.44 mm, and a magnification of 3,000×. The prepared patterned PG hydrogels were stored in a refrigerator at 4 °C, and when it is time to test, it is removed and the surface is sprayed with gold and then observed using SEM for visual analysis.

#### Tensile test

The PG hydrogels, prepared in advance, were sliced into elongated strips measuring 20 mm in length, 4 mm in width, and 0.15 mm in thickness. Subsequently, the stress–strain characteristics of these strips were evaluated at room temperature using an electromechanical universal testing machine (CMT2103).

#### Contact angle

The contact angle of water on the surface of the PG patterned hydrogel was measured with a JY-82B contact angle analyzer (KRUSS, Germany).

#### Stability of pattern topography

The stability of the micropattern on the PG hydrogel was evaluated by PBS immersion. The hydrogels were placed into PBS at 37 °C. Photos were taken at 1, 3, 7, 14, 21, and 30 d of incubation. The samples were then removed, and the micropattern was observed under an optical microscope.

### Cell culture and seeding

Primary cultures of RTFs were established by transplanting from the tenon tissue of rabbits. The tenon tissue was taken and washed repeatedly with PBS supplemented with 1% penicillin–streptomycin–gentamicin solution 3 times and then cut into pieces of 0.5 to 1 mm^3^ size in fetal bovine serum (FBS). Tissue fragments ZRTFs from two to five passages were used in this study. Myofibroblast activation was induced using 5 ng/ml TGF-β.

### In vitro biocompatibility

The PG hydrogels were cut into a uniform size of 3 mm in diameter and 1 mm in height round shape. Prior to cell culture, materials were sterilized with penicillin–streptomycin–gentamicin.

RTFs and RAWs were seeded in 24-well plates separately. After being cultured for 1 d, transwells containing silicon or PG hydrogels were put into the 24-well plate and then cultured for 5 d. The cytotoxicity evaluation of silicon and PG hydrogels was evaluated by CCK-8 reagent. Starting from the first day, the optical density was measured every 2 d (1, 3, and 5). The cytotoxicity of each group was calculated by the absorbance.

Live/dead cells were quantified using Calcein/PI Kit staining. After incubation with the staining liquor at 37 °C for 30 min, samples were observed by fluorescence microscopy (Leica, Germany).

### Cell proliferation on hydrogels

RTFs were seeded on silicon, flat gels, and microgroove widths of 5-, 10-, 15-, and 20-μm pattern gels into 24-well plates with 1 × 10^5^ cells/well. After incubating for target date, CCK-8 reagent was used to detect cell proliferation.

### Immunofluorescence

Cells or tissues were fixed for 15 min with 4% paraformaldehyde (PFA), permeabilized with 0.2% Triton X-100 15 min, and incubated with 5% bovine serum albumin (BSA) for 1 h, and primary antibody was then added overnight at 4 °C. Fluorescent secondary antibody was added at 37 °C. Images were captured randomly in three fields of each sample. Cytoskeletal immunostaining was assessed by tetramethyl rhodamine isothiocyanate (TRITC)-conjugated phalloidin staining.

### Real-time PCR

Total RNA was extracted, and cDNA was synthesized. Three replicates of the SYBR Green (Roche, Switzerland) reaction mixture were added to eight tube strips, followed by quantitative PCR analysis.

### Western blot analysis

The concentration of the extracted protein samples was determined by a silver cholate kit, followed by gel electrophoresis using three replicate samples. The gel was then transferred to the membrane and then blocked and incubated with α-SMA primary antibody. Fluorescent secondary antibody was added at 37 °C for 1 h and visualized by chemiluminescence.

### Experimental animals and implantation surgeries

For this study, 60 New Zealand white rabbits (12 to 14 weeks, 2 to 3 kg) were acquired from Laboratory Animal Research Center at the Second Affiliated Hospital of Harbin Medical University. In vivo experiments were divided into four groups, namely, commercially available AGV FP8 (control group), MMC-loaded AGV FP8 (MMC group), flat PG (flat GDD group), and patterned PG (pattern GDD group), with an implantation number of *n* = 6 in each group. The implanted GDD was fabricated by combining the AGV valve structure and hydrogel. Either eye was selected for surgery. The number of rabbits was chosen based on our previous studies with a similar animal model.

Sumianxin II (0.2 ml/kg) was administered intramuscularly, followed by intravenous anesthesia of 3% pentobarbital sodium (1.2 ml/kg). The eyes were wiped and rinsed twice with povidone–iodine solution. After injecting 2% lidocaine at the surgical site to achieve local anesthesia, we made a parallel incision and separated the conjunctiva from the sclera bluntly. Then, we brought the implants to the surgical site, secured the drainage tube on the scleral surface, primed the valve with a rinse needle and balanced salt solution irrigation, made a scleral tunnel with a 23-gauge needle, and inserted the tube into the tunnel. Finally, the conjunctiva was secured to the corneal limbus.

### Postoperative clinical observation

General health observations were performed daily. Ofloxacin eyedrop was given to the operated eyes three times a day for 1 week after the operation. IOP was recorded by the same doctor under superficial anesthesia with a Tono-Pen tonometer (Reichert Technologies, NY) before surgery and on days 3, 5, 7,14, 30, 60, and 90 following surgery. The corneal situation, central anterior chamber depth, conjunctiva thickness, and unobstructed rate of the tube were evaluated by a highly experienced doctor using AS-OCT on days 14, 30, 60, and 90 following surgery. These time points conformed to the standard of care in similar studies [6,12]. To analyze scan sections at the same position for each inspection, we measured the bleb wall thickness at the point 2 mm from the bleb edge. Images were analyzed by the same observer.

### Histopathological examination

Upon completing the in vivo observations, rabbits were euthanized by overdose anesthesia (2 ml/kg 3% pentobarbital sodium). The eyes were enucleated carefully without disturbing the blebs and immediately immersed in 4% PFA for 3 d at 4 °C. Tissues were dehydrated, embedded in paraffin, and then sent for sequential pathological sections (4 μm thickness). Samples were photographed by optical microphage in 3 sections along the implant fibrosis tissue periphery.

### Statistical analysis

Statistical analyses were conducted using GraphPad Prism 9. Data are presented as means ± SD. The Student’s *t* test and one-way analysis of variance (ANOVA) were used for statistical evaluation. *P* values less than 0.05 were considered statistically significant. Symbols *, **, and *** indicate *P* < 0.05, 0.01, and 0.001, respectively.

ImageJ was employed to analyze cell number, area, fluorescence intensity, and calculated regions positive for collagen, myofibroblast activation, and collagen I/III relative expression of the bleb capsule.

## Ethical Approval

Animal experiments are necessary to observe the anti-fibrosis effect of patterned GDDs in GDD implantation. All animal study protocols were approved by the Animal Ethics Committee of the Second Affiliated Hospital of Harbin Medical University (YJSDW2023-085). Adult New Zealand white rabbits housed under standard conditions were used in this study. All experiments followed the Association for Research in Vision and Ophthalmology (ARVO) Statement for the Use of Animals in Ophthalmic and Vision Research. Rabbits were anesthetized by intravenous injection of 1.2ml/kg 3% pentobarbital sodium. Rabbits were euthanized by overdose anesthesia (2 ml/kg 3% pentobarbital sodium). The authors have adhered to the ARRIVE guidelines.

## Data Availability

The data that support the findings of this study are available from the corresponding author upon reasonable request.
