# Supplementary material for: Anti-Scar Effects of Micropatterned Hydrogel after Glaucoma Drainage Device Implantation
Source: Research (Wash D C). 2025 Jan 22;8:0561. doi: 10.34133/research.0561 (PMC11751202; doi:10.34133/research.0561)
Supplement: Supplementary 1 — Figs. S1 to S3 [file research.0561.f1.zip › Supplementary Information.docx]

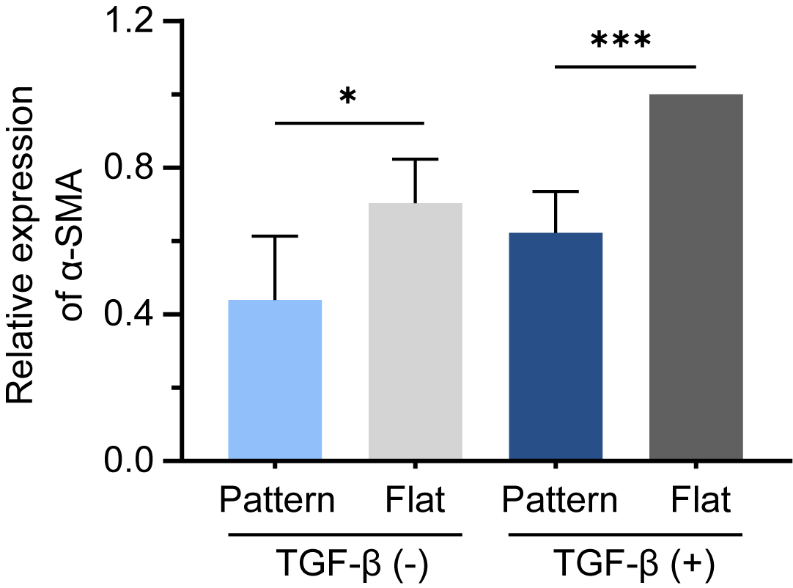


**Fig. S1.** Western blot analysis of α-SMA expression.


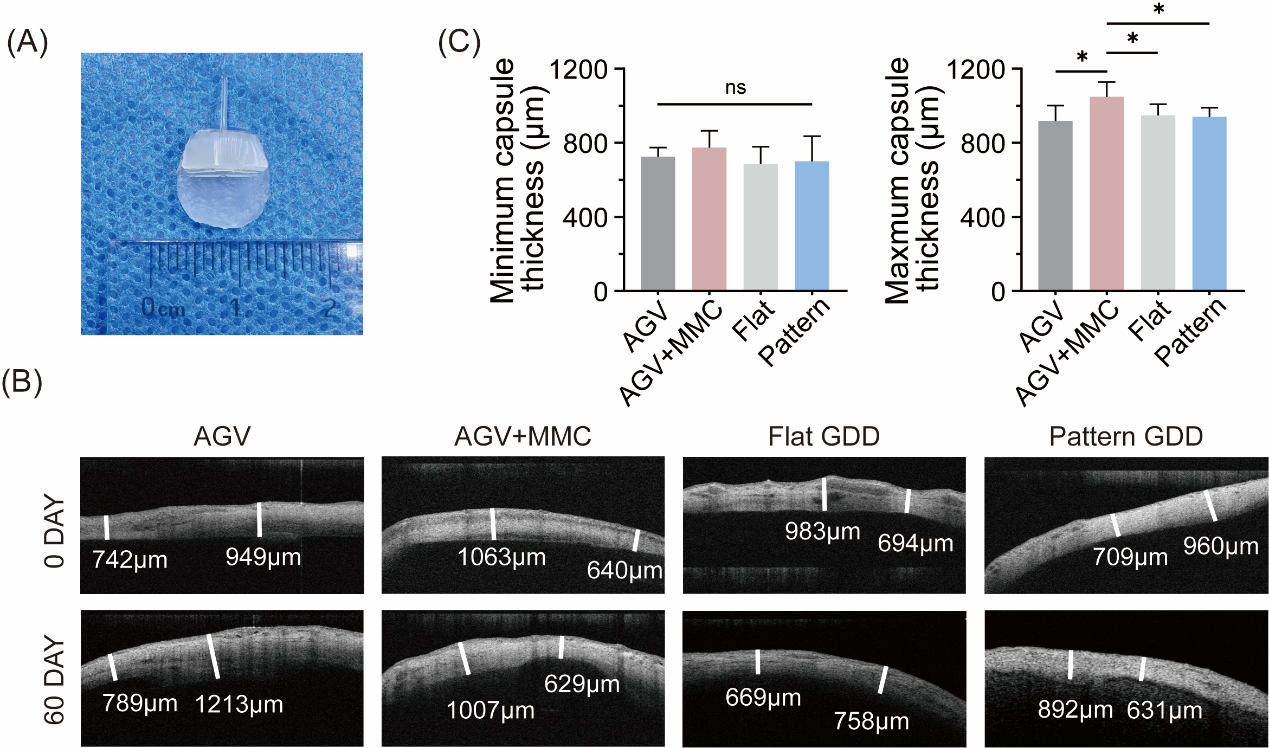


**Fig. S2.** (A) Image of the produced GDDs. (B) AS-OCT images of the filter bleb at 0 and 60 days postoperation. (C) Maximum and minimum capsule thickness of filter bleb at 0 days postoperation.


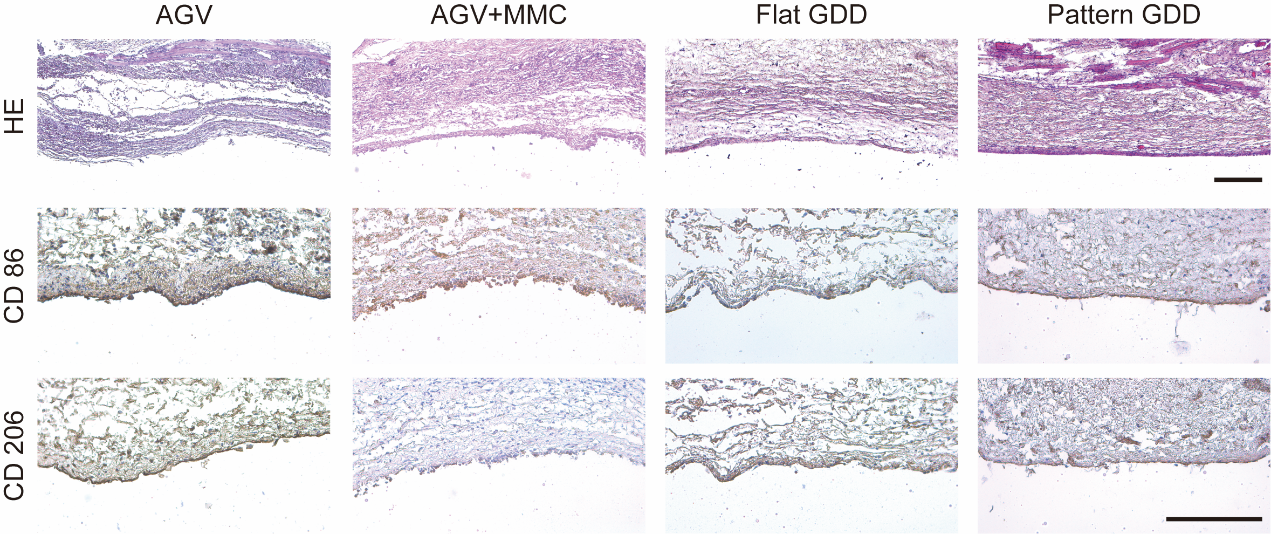


**Fig. S3.** HE and IHC staining of conjunctiva for CD86 (M1 phenotype), CD206 (M2 phenotype) at 4 days post-operation. Scar bar:100 μm.
